# Chalcone-derivative L6H21 attenuates the OVA-induced asthma by targeting MD2

**DOI:** 10.1186/s40001-023-01630-5

**Published:** 2024-01-20

**Authors:** Xiangting Ge, Tingting Xu, Meiyan Wang, Lijiao Gao, Yue Tang, Ningjie Zhang, Rui Zheng, Weimin Zeng, Gaozhi Chen, Bing Zhang, Yuanrong Dai, Yali Zhang

**Affiliations:** 1 Department of Pulmonary and Critical Care Medicine, The Second Affiliated Hospital and Yuying Children’s Hospital of Wenzhou Medical University, Wenzhou, 325000 Zhejiang China; 2 Affiliated Yueqing Hospital, Wenzhou Medical University, Wenzhou, 325600 Zhejiang China; 3 Chemical Biology Research Center, School of Pharmaceutical Sciences, Wenzhou Medical University, Wenzhou, 325035 Zhejiang China; 4 Division of Pulmonary Medicine, Key Laboratory of Heart and Lung, the First Affiliated Hospital of Wenzhou Medical University, Wenzhou, 325000 Zhejiang China

**Keywords:** Chalcone derivative, MD2 inhibitor, TLR4, MD2 recombination, Asthma

## Abstract

Asthma represents a significant global challenge that affects individuals across all age groups and imposes substantial social and economic burden. Due to heterogeneity of the disease, not all patients obtain benefit with current treatments. The objective of this study was to explore the impact of MD2 on the progression of asthma using L6H21, a novel MD2 inhibitor, to identify potential targets and drug candidates for asthma treatment. To establish an asthma-related murine model and evaluate the effects of L6H21, ovalbumin (OVA) was used to sensitize and challenge mice. Pathological changes were examined with various staining techniques, such as H&E staining, glycogen staining, and Masson staining. Inflammatory cell infiltration and excessive cytokine secretion were evaluated by analyzing BALF cell count, RT-PCR, and ELISA. The TLR4/MD2 complex formation, as well as the activation of the MAPK and NF-кB pathways, was examined using western blot and co-IP. Treatment with L6H21 demonstrated alleviation of increased airway resistance, lung tissue injury, inflammatory cell infiltration and excessive cytokine secretion triggered by OVA. In addition, it also ameliorated mucus production and collagen deposition. In the L6H21 treatment group, inhibition of MAPK and NF-кB activation was observed, along with the disruption of TLR4/MD2 complex formation, in contrast to the model group. Thus, L6H21 effectively reduced the formation of the MD2 and TLR4 complex induced by OVA in a dose-dependent manner. This reduction resulted in the attenuation of MAPKs/NF-κB activation, enhanced suppression of inflammatory factor secretion, reduced excessive recruitment of inflammatory cells, and ultimately mitigated airway damage. MD2 emerges as a crucial target for asthma treatment, and L6H21, as an MD2 inhibitor, shows promise as a potential drug candidate for the treatment of asthma.

## Introduction

Asthma refers to a heterogeneous disease affecting all age groups, characterized by airway hyperresponsiveness (AHR) and chronic airway inflammation [22]. This global issue affects a worldwide population of 339 million, and it is projected to increase to 400 million by 2025 [8]. It is important to understand the fundamental mechanism of asthma and explore new therapeutics to curtail the increasing incidence and burden of asthma. Corticosteroids, as classic anti-inflammatory drugs, are critical to treat acute and chronic airway inflammatory diseases, such as asthma [6, 27, 43]. The current asthma therapy predominantly relies on bronchodilators and inhaled corticosteroids, which serve to alleviate symptoms but do not modify the natural course of asthma. Unfortunately, this approach falls short in effectively managing symptoms for 5–10% of patients [35]. Furthermore, treatment with the use of corticosteroids in the long run generally leads to some side effects [19, 44]. Hence, it is essential to understand the underlying causes of asthma and explore alternative, more effective therapeutic options.

According to the classic Th1/Th2 lymphocyte imbalance theory, Th2 cells play a dominant role in asthma [28]. Particularly, activated Th2 cells or ILC2s in Th2-high asthma drive chronic airway inflammation. Type 2 cytokines (e.g., IL-13, IL-5, and IL-4) facilitate asthma’s hallmark characteristics (e.g., susceptibility to exacerbations, IgE production, AHR, IgE production, mucus hypersecretion, and eosinophilia [15, 25]). Nevertheless, asthma is complicated and exhibits heterogeneity. Some asthmatic patients can be treated by targeting molecular mediators of type 2 immunity [3, 34]. One limitation in the use of these biologics is that specific populations of patients should be carefully selected based on phenotypes and endotypes of asthma [34].

Myeloid differentiation protein 2 (MD2) and toll-like receptor 4 (TLR4) are essential components of the innate immune system, serving as pattern recognition receptors and co-receptors, respectively, in the recognition of pathogenic microorganisms and initiation of immune responses [2]. Upon transport to the cell membrane surface, lipopolysaccharide (LPS) directly binds to MD2, forming the MD2–LPS complex. This further binds to TLR4 to form the LPS–MD2–TLR4 ternary complex, dimerizes the ternary complex to enable TLR4 to penetrate the membrane and alter the intramembranous structure and conformation, and therefore activates the TLR4 downstream-signaling pathway in innate immunity. The downstream signaling of TLR4 is majorly composed of the myd88-dependent MAPKs/NF-κB transcription pathway and the TRIF-dependent interferon-beta (IFN-β) transcription pathway, which turn on pro-inflammatory genes’ transcription and expression (e.g., chemokines, adhesion molecules, as well as inflammatory cytokines) [7]. MD2 is the "first gateway" for the recognizing and binding of LPS, taking on a great significance to TLR4-mediated LPS recognition and inflammatory signal transduction [47]. TLR4’s expression is found from many cells, includes airway smooth muscle cells [23] and airway epithelial cells [45]. TLR4 knockout mice and MyD88 knockout mice significantly down-regulate allergen-induced Th2 inflammatory cytokines’ expression (IL-4, IL-5, IL-13) and improve AHR [14, 33], suggesting that allergens may mediate airway inflammation through the TLR4–MyD88-signaling pathway. Smit et.al found that the association between occupational endotoxin exposure and wheeze in agricultural workers was significantly modified by genetic variants in MD2 [41]. However, there is little basic research on the role of MD2, an essential helper protein of TLR4, in asthma.

Chalcone, a naturally occurring compound, possesses diverse biological activities, including anti-inflammatory and anti-tumor properties [39]. Our research group has conducted extensive investigations into the pharmaceutical chemistry and chemical biology of chalcone, leading to the identification of several natural chalcone compounds exhibiting remarkable anti-inflammatory effects. Notably, one such compound, L6H21 (Fig. 2A), has demonstrated significant inhibition of the release of inflammatory factors induced by LPS [49]. Furthermore, our research has revealed that the structure of L6H21 bears similarity to MD2 inhibitors, and through multiple tests, L6H21 has been identified as a novel MD2 inhibitor [46]. In this study, we aim to investigate whether L6H21 can alleviate inflammation and pathophysiological changes associated with asthma, while also exploring the role of MD2 in the progression of asthma.

## Materials and methods

### Reagents

MD2 antibody used for Immunohistochemistry was from Abcam (Cambridge, UK). Cell-Signaling Technology (Danvers, MA, USA) provided antibodies in terms of GAPDH, p-JNK, JNK, p-P38, and P38. Invitrogen (Carlsbad, CA, USA) offered Mouse IL-6, TNF-α, and IgE ELISA kits. MD2 antibody used for WB was from eBioscience (San Diego, CA, USA). R&D Systems (Minneapolis, MN, USA) provided recombinant human MD2 (rhMD2) protein. Santa Cruz Biotechnology (Dallas, TX, USA) offered antibodies in terms of ERK, p-ERK, IκB, and TLR4.

### Immunohistochemistry of human samples

Samples of human lung tissue slices from two asthma patients and two non-asthma individuals were obtained from the peripheral lung tissues of patients undergoing pulmonary nodule resection at the Second Affiliated Hospital of Wenzhou Medical University, and provided signed informed consent. These samples were used to perform immunohistochemistry for MD2 using routine techniques. Sections were then deparaffinized, rehydrated, treated with 3% H2O2 for 30 min to block endogenous peroxidase activity, and blocked with 5% goat serum for 1 h after antigen retrieval. Slides were incubated overnight at 4 °C with primary antibodies, and immunoreactivity was detected by diaminobenzidine (DAB). Slides were counterstained with hematoxylin for 100 s, dehydrated, and mounted for viewing by bright-feld microscopy (Nikon, Japan).

### Experimental animals

Wenzhou Medical University Animal Center provided C57BL/6 mice (male, 6–8 weeks). Animals were housed and given water and a diet that met the criteria of rodents on in accordance with a 12:12-h light–dark cycle within the Specific Pathogen Free (SPF) grade animal room.

### OVA-triggered asthmatic mouse model

Mice were randomly assigned a treatment group using L6H21 at a high dose (20 mg/kg) [OVA + L6H21(20)], a treatment group using L6H21 at a low dose (10 mg/kg) [OVA + L6H21(10)], an OVA group (OVA), a L6H21(20 mg/kg) group (L6H21), and a control group (CON). The respective group comprised six mice. We set the animal model of asthma as previously described [51]. In brief, a suspended mixture of Al(OH)3 (2 mg, Sigma-Aldrich, St Louis, MO, USA) and OVA (20 μg, Sigma-Aldrich, St Louis, MO, USA) within saline with a volume of 200 μL was injected on day 0 and day 14 intraperitoneally to sensitize the L6H21 and OVA treatment groups (both L6H21 at a small dose and a great dose). OVA (1% in saline) aerosol was utilized to challenge the OVA and L6H21 treatment groups from day 25 to day 31 for 40 min per day. Saline with an equal volume was adopted to administer the control group and L6H21 group through intraperitoneal injection on day 0 and day 14. Aerosolized saline was used to treat these groups for 40 min each day from day 25 to day 31. Since day 25, half an hour before being aerosolized per day, the mice had been subjected to 0.5% sodium carboxymethyl cellulose solution treatment or 10 mg/kg or 20 mg/kg L6H21 treatment (in 0.5% sodium carboxymethyl cellulose solution) on the basis of gavage (Fig. 2B). We examined AHR and acquired serum, bronchoalveolar lavage fluid (BALF) and tissues for in-depth investigation 24 h after the last challenge was completed (day 31).

### Measurement of AHR

80 mg/kg pentobarbital (Sigma-Aldrich, St. Louis, MO, USA) was adopted to anesthetize the mice through intraperitoneal injection 24 h after the final OVA challenge. Then, to achieve mechanical ventilation, mice were subjected to tracheostomy (18-gauge cannula); for AHR evaluation, flexiVent SCIREQ (Montreal, Quebec, Canada) was adopted to rapidly connect the mice [1]. 0.25 mL tidal volume was employed for mechanical ventilation of mice for mimicking spontaneous ventilation (under 3 cm H_2_O positive end-expiratory pressure and 150 breaths/min). Methacholine (Mch, Sigma-Aldrich, St Louis, MO, USA) at rising concentrations (3.125, 6.25, 12.5, 25, and 50 mg/mL) and saline aerosol were adopted to challenge the mice for 10 s at 4–5 min intervals when baseline was completely measured. Data on airway resistance were continuously collected. For calculating airway dynamic compliance, we adopted the average of the three maximal values.

### BALF acquisition and cytology

Thoracotomy and ligation of the right lung were performed. 200μL PBS was adopted to infuse the left lung quartic through the tracheal cannula to obtain BALF as previously described [54]. BALF were centrifugated at 3000 × rpm at 4 °C. The cell-free supernatant was immediately stored at −80 °C. Target cytokines were measured in the supernatant. 50 μL PBS was adopted to resuspend the cell pellets from the BALF. Cells’ total number in the BALF was acquired with the use of Count Star (Shanghai, China). Then, we examined the number of eosinophils in the BALF via counting at least 200 cells per slide through Wright–Giemsa staining (Jiancheng, Nanjing, China).

### ELISA

According to the manufacturer's instructions, we utilized commercial ELISA kits to quantify the levels of interleukin-6 (IL-6), TNF-α in BALF, and IgE in serum. Briefly, we coated an ELISA plate with monoclonal capture antibodies and subsequently added 100 μL of serum/BALF supernatant or gradient standard following the 1-h blocking step using ELISPOT diluent. The plate was then incubated at room temperature for 2 h. After five washing steps, monoclonal detection antibodies conjugated with horseradish peroxidase were added to each well, followed by a 1-h incubation at room temperature. Subsequently, the plate was washed, and tetramethylbenzidine was added to each well. The reaction was stopped using 2 M H_2_SO_4_. The absorbance at wavelengths of 450 nm and 570 nm was measured using a SpectraMax M5 plate reader (Molecular Devices, Sunnyvale, CA, USA).

### Histopathological study

4% Paraformaldehyde was used to fix the middle lobe of the right lung. Next, the lobe was immersed in paraffin. The lung sections (5 μm) were dehydrated and stained with Masson trichrome to achieve collagen deposition, Periodic Acid Schiff (PAS) to achieve mucus secretion, and hematoxylin and eosin (H&E) to achieve inflammation. Regarding lung inflammatory score, a 5-point scoring system were used: 0, no inflammatory cells were identified; 1, bronchi were surrounded by more than 5 layer of inflammatory cell; 2, bronchi were surrounded by 2–4 layer of inflammatory cell; 3, bronchi were surrounded by 1 layer of inflammatory cells; and 4, inflammatory cells were occasionally identified. The mucus scores in each airway were determined in the following: 4, > 75% PAS-positive cells; 3: 50–75% PAS-positive cells; 2, 25–50% PAS-positive cells; 1, < 25% PAS-positive cells; 0, no PAS-positive cells. We determined the scores of inflammatory cells and PAS-positive cells in no less than three different airways for the respective tissue section. We determined the mean scores. Slides were scored blindly by two independent investigators.

### Real-time quantitative polymerase chain reaction (RT-qPCR)

Total RNA was extracted from lung tissues (10–20 mg) with the use of TRIzol-reagent (Invitrogen, Carlsbad, CA, USA). Ultraviolet (UV) absorption at 260 and 280 nm was adopted to estimate the RNA concentration and quality. M-MLV Platinum RT-qPCR Kit (Invitrogen, Carlsbad, CA, USA) was adopted to performing reverse transcription and quantitative PCR. We carried out RT-qPCR analysis using a Mastercycler ep RealPlex detection system (Eppendorf, Hamburg, Germany). Data were obtained with the use of the 2^−ΔΔCt^ method. The primers of genes including β-actin, collagen IV, TGF-β, TNF-α, IL-6, IL-13, IL-5, and IL-4 were obtained from Sangon (Shanghai, China). Table 1 lists the sequences of primers employed. β-Actin served as the internal reference.

**Table 1:** Primers used for real-time qPCR assay

Gene	Species	Forward primer	Reverse primer
β-actin	Mouse	CCGTGAAAAGATGACCCAGA	TACGACCAGAGGCATACAG
Collagen IV	Mouse	TGGCCTTGGAGGAAACTTTG	CTTGGAAACCTTGTGGACCAG
IL-4	Mouse	GGAACAAAGACCTGTGGGGT	GGCATCGAAAAGCCCGAAAG
IL-5	Mouse	TCCTCTTCGTTGCATCAGGG	TGTGGCTGGCTCTCATTCAC
IL-6	Mouse	GAGGATACCACTCCCAACAGACC	AAGTGCATCATCGTTGTTCATACA
IL-13	Mouse	TGCCATCTACAGGACCCAGA	CTCATTAGAAGGGGCCGTGG
TGF-β	Mouse	TGACGTCACTGGAGTTGTACGG	GGTTCATGTCATGGATGGTGC
TNF-α	Mouse	TGATCCGCGACGTGGAA	ACCGCCTGGAGTTCTGGAA

### Western blotting assay analysis

Total protein of mouse lung tissue was extracted with the use of RIPA lysis buffer (BOSTER, Wuhan, China) supplemented with protease inhibitor and phosphatase inhibitors for obtaining protein. BCA protein assay kit (Beyotime, Shanghai, China) was adopted to quantify total proteins’ concentration. For Western blotting assay, the equal amounts of protein (60 μg) were electrophoresed in 10% sodium dodecyl sulfate–polyacrylamide gel (SDS–PAGE) and electro-transferred onto a polyvinylidene fluoride membrane (Bio-Rad, Hercules, CA, USA). Blocking buffer (5% non-fat milk in Tris-buffered saline supplemented with 0.1% Tween 20 [TBST]) was adopted for 1.5-h blocking of non-specific binding at ambient temperature. Subsequently, specific primary antibodies were employed to incubate the membranes throughout the night at 4 °C. The membranes were reacted for 1 h at ambient temperature with corresponding secondary horseradish peroxidase-conjugated antibody (Santa Cruz, CA, USA; 1:3000) following three washing processes using TBST. Finally, immunoblot signals were visualized with the use of enhanced chemiluminescence reagents (Bio-Rad, Hercules, CA, USA). ImageJ software (NIH, Bethesda, MD, USA) was adopted to quantifying immunoreactive bands’ relative intensities.

### Co-immunoprecipitation assay

Protein samples from Lung tissue were subjected to 10-min centrifugation at 12000 × rpm at 4 °C. In 300 μg of protein, we added enough MD2 antibody. Samples received the gentle rotation at 4 °C throughout the night. Protein A + G agarose was adopted to collect the immune complexes. Ice-cold PBS was used to wash the precipitates four times. Then, the proteins were released by boiling them in a sample buffer. Next, Western blotting analysis was carried out following the above description.

### Statistical analysis

We adopted GraphPad Prism 9.0 software to carry out statistical analysis. Data have the expression of the mean ± standard error of measurement (SEM). The difference of data sets was studied through Student’s *t* test or one-way ANOVA, as well as Dunn’s post hoc test. A *p* value less than 0.05 indicated statistical significance, denoted as *.

## Results

### MD2 expression in asthmatic patients is elevated

**Fig. 1:**
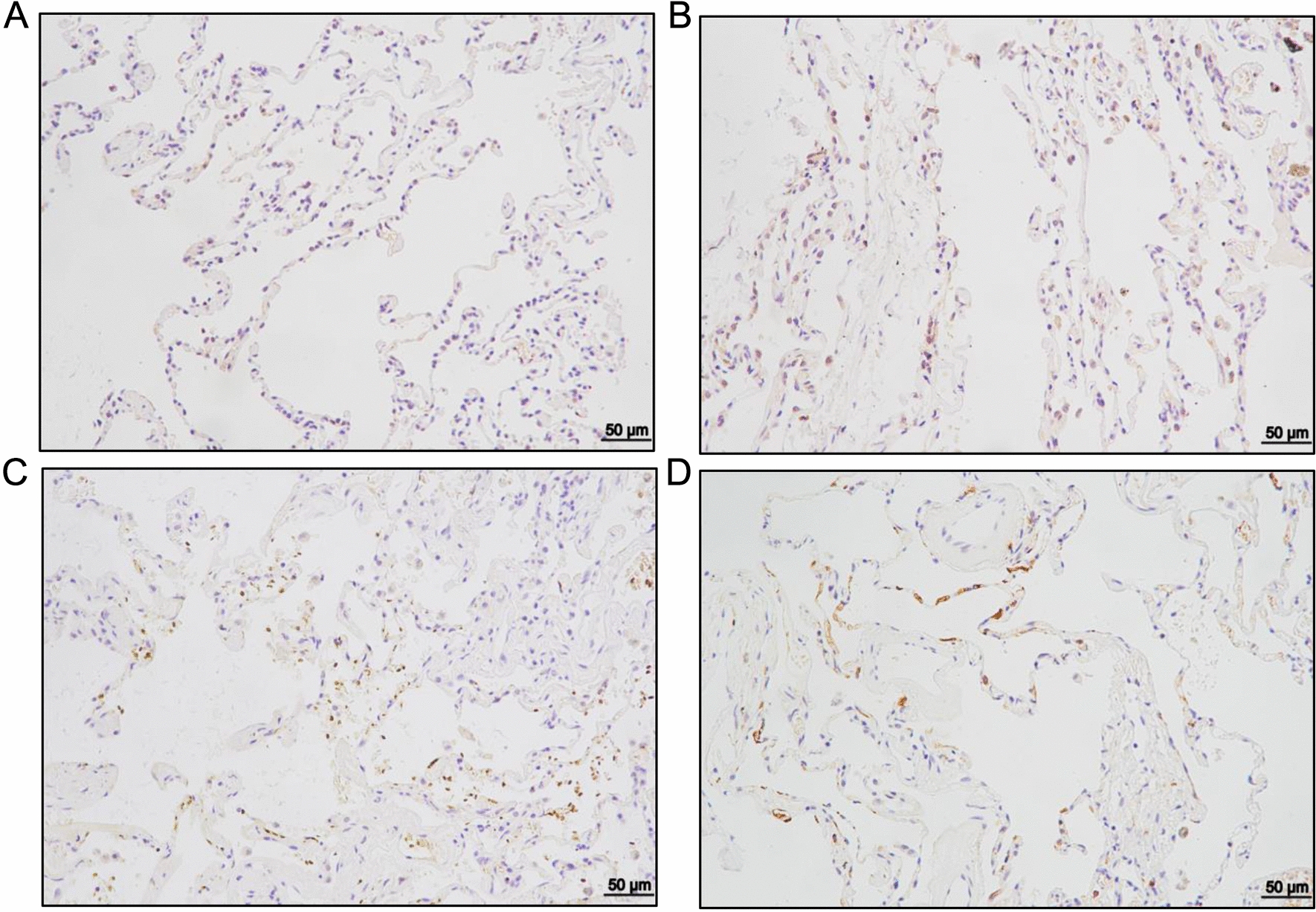
MD2 expression in asthmatic patients is elevated than those in non-asthmatic patients. immunohistochemistry for MD2 (200X) **A** Non-asthmatic patient A, **B** non-asthmatic patient B, **C** asthmatic patient A, **D** asthmatic patient B

First, to preliminarily explore the clinical relevance of MD2 to asthma, we investigated whether there were expression differences of MD2 between asthma patients and non-asthma patients. Immunohistochemistry revealed that the expression level of MD2 in lung tissue of asthma patients was higher than that in non-asthma patients (Fig. 1), suggesting that MD2 as a potential target for asthma treatment has important clinical significance.

### L6H21 relieves OVA-triggered AHR in mice

**Fig. 2:**
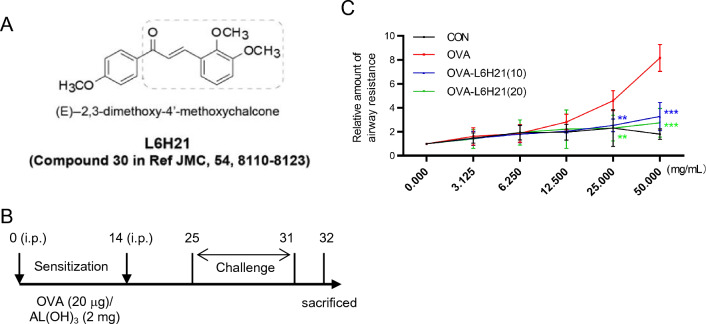
L6H21 relieving OVA-triggered AHR in mice. **A** Chemical structure of L6H21. **B** Experimental schemes of mouse asthma model. OVA/Al(OH)_3_ was adopted to sensitized mice on day 0 and day 14; from day 25 to day 31, 1% OVA aerosol was used to expose the mice for 7 continuous days. **C** Airway resistance in mice. According to the evaluation, airway responsiveness indicated the mean response of mice with mechanical ventilation to risen Mch doses (mean ± SEM; *n* = 6 in the respective group; **P < 0.01 in comparison with the OVA group)

An OVA-induced asthma model was established to evaluate the effect of MD2 inhibitor L6H21 on it. AHR is defined as an excessive airway smooth muscle contraction to various stimuli, which is a major pathophysiological characteristic and diagnosis criteria of asthma [50]. It has been reported that OVA sensitize and challenge can induce mice develop AHR, as measured by airway resistance (Rn) after Mch challenges [48]. In our study, AHR, which is typically reflected by high Rn, was identified in the OVA group. However, the Rn of L6H21 treatment group, both 10 mg/kg and 20 mg/kg showed significantly decreased (Fig. 2C).

### L6H21 attenuates OVA-triggered damage of lung tissues and inflammatory cells recruitment

**Fig. 3:**
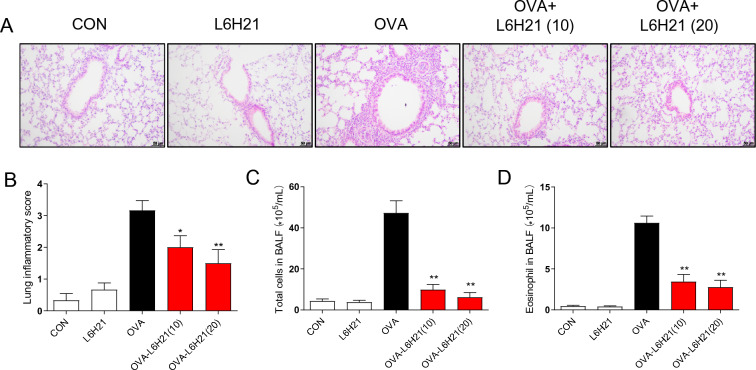
L6H21 attenuated OVA-triggered histopathologic changes and inflammatory cell recruitment. **A** H&E staining(200X) was performed to identify inflammatory cell infiltration and tissue damage of the lung in mice. **B** Histological analysis was conducted on lung tissues to calculate lung inflammatory score. **C**, **D** After the OVA challenge was completed, L6H21 decreased eosinophils (**D**) and total cells (**C**) within BALF (results have an expression of the mean ± SEM; *n* = 6 in the respective group; *P < 0.05, **P < 0.01 in comparison with the OVA group)

To investigate the contribution of L6H21 on OVA-triggered damage of lung tissues and airway inflammatory cell recruitment, H&E staining was conducted. As illustrated in Fig. 3A, OVA-challenged mice exhibited significantly inflammatory cell infiltration**,** thickened airway walls, confined lumens, and shed tracheal epithelial cells compared to these in control group and L6H21 group. L6H21 treatment relieved these pathologic changes, with reduced histological inflammation score (Fig. 3B).

Total cells and eosinophils in BALF were determined by cytometer and Wright–Giemsa staining, respectively, to evaluate inflammatory cell recruitment. As expected, the numbers of total inflammatory cells (Fig. 3C) and eosinophils (Fig. 3D) in BALF were remarkably increased in OVA group, while significantly abrogated by L6H21 treatment. These data suggest that L6H21 was able to mitigate high inflammation status in asthmatic mice.

### L6H21 reduces OVA-triggered serum IgE and Th2 cytokine levels

**Fig. 4:**
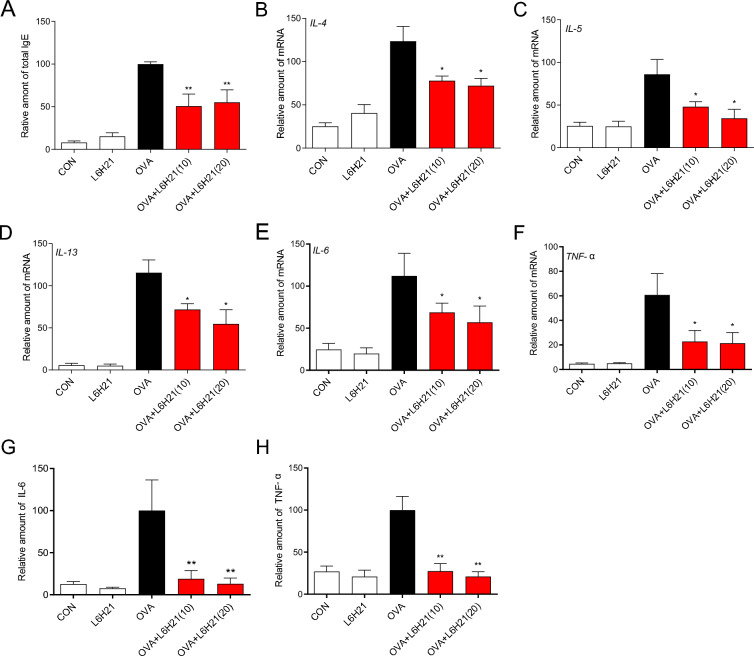
L6H21 attenuated OVA-triggered IgE, Th2 cytokines, and inflammatory factor secretion. **A** L6H21 ameliorate OVA-triggered increase in IgE in serum. **B**, **C** Effects of L6H21 on attenuating OVA-triggered increase in IL-4 (B), IL-5 (**C**) and, IL-13 (**D**) in BALF. **E**, **F** RT-qPCR was performed to examine TNF-α (**F**) and IL-6 (**E**) mRNA level in lung tissue. **G**, **H** ELISA was adopted to examine TNF-α (**H**) and IL-6 (**G**) protein level in BALF (results have an expression of the mean ± SEM; *n* = 6 in the respective group; *P < 0.05, **P < 0.01 in comparison with the OVA group)

The crucial role of IgE and Th2 cytokines in the development of airway inflammation associated with allergies is widely recognized [12]. Therefore, the protein expression levels of total IgE and the gene expression levels of Th2 cytokines (IL-4, IL-5, and IL-13) were measured in serum and lung tissues, respectively, for verifying L6H21’s effects in vivo. We identified a markedly elevated serum IgE in OVA-challenged mice, while it was robustly diminished after oral administration of L6H21, even at a low dose (10 mg/kg, Fig. 4A). Similar results were identified in the mRNA expression of IL-13, IL-5, and IL-4 in lung tissues (Fig. 4B–D).

### L6H21 suppresses OVA-triggered inflammatory factor secretion

Accumulating evidence suggest that combined pharmacological inhibition of pathogenic cytokines (e.g., IL-6 and TNF-α) is more effective than single Th2 cytokine targeting in some cases [11, 26]. Based on these findings, the gene and protein expression levels of TNF-α and IL-6 in lung tissues and BALF, respectively, were measured. As presented in Fig. 4E, F, the mRNA expression level of TNF-α and IL-6 in mice exposed to OVA was 5–10 times higher than that exposed to normal saline, and no significant differences in TNF-α and IL-6 levels were found among control group and L6H21 group. Asthmatic mice treated with L6H21 showed powerful suppression of TNF-α and IL-6 expression, which was dependent on concentration of L6H21. The same change can be identified in the TNF-α and IL-6 protein levels in BALF (Fig. 4G, H).

### L6H21 attenuates OVA-triggered mucus production and collagen deposition

**Fig. 5:**
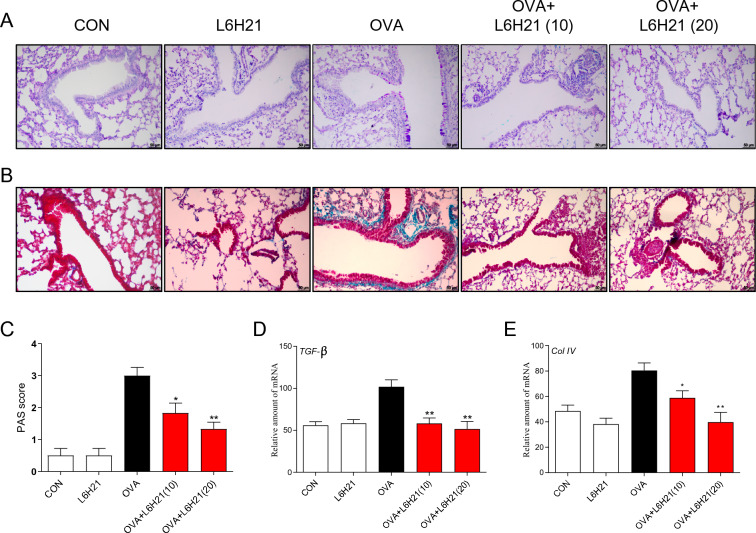
L6H21 attenuated OVA-triggered goblet cell hyperplasia and airway remodeling. **A** Goblet cell hyperplasia in the airway epithelium was identified using PAS staining (200X). **B** Masson staining (200X) was performed to identify collagen deposition of the airway in mice. Goblet cell scores **C** were evaluated by histological analysis of lung tissues. **D**, **E** Relative mRNA level of TGF-β (**D**) and Col IV (**E**) were examined through RT-qPCR (results have an expression of the mean ± SEM; *n* = 6 in the respective group; *P < 0.05, **P < 0.01 in comparison with the OVA group)

Goblet cell formation, known as the key feature of asthma and the source of mucus production, can be identified even in some patients with newly diagnosed asthma [38]. We tested whether L6H21 could inhibit OVA-triggered goblet cell formation by PAS staining. Increased goblet cell proliferation and PAS score could be identified in OVA-challenged mice than the control group and L6H21 group. However, even low dose L6H21 treatment could significantly curtail the goblet cell formation and PAS score (Fig. 5A, C).

Airway smooth muscle cells are stimulated to release TGF-β, promote extracellular matrix (ECM) secretion and collagen synthesis [13, 20, 36], eventually driving airway fibrosis in the asthma model. Therefore, Masson trichrome staining was used to detect collagen deposition in mice. OVA challenge caused increased collagen deposition in the lung interstitium around airways and blood vessels in comparison with control mice as shown by Masson staining, while the amount of collagen surrounding airways and blood vessels was markedly reduced by L6H21 administration (Fig. 5B). Considering that Collagen IV and TGF-β play an essential role in collagen deposition, the mRNA expression of TGF-β and collagen IV was determined. RT-qPCR revealed a significant increase in mRNA of TGF-β and collagen IV in asthmatic mice lung tissues in comparison with those in control group and L6H21 group. Nonetheless, oral administration of L6H21 (both 10 mg/kg and 20 mg/kg) drastically decreased these elevations (Fig. 5D, E). These data demonstrated that L6H21 down-regulated TGF-β and collagen IV expression induced by OVA, restrained ECM deposition and collagen synthesis, result in the suppression of airway fibrosis.

### L6H21 inhibits OVA-triggered MAPK and NF-кB pathways activation in allergic airway inflammation

**Fig. 6:**
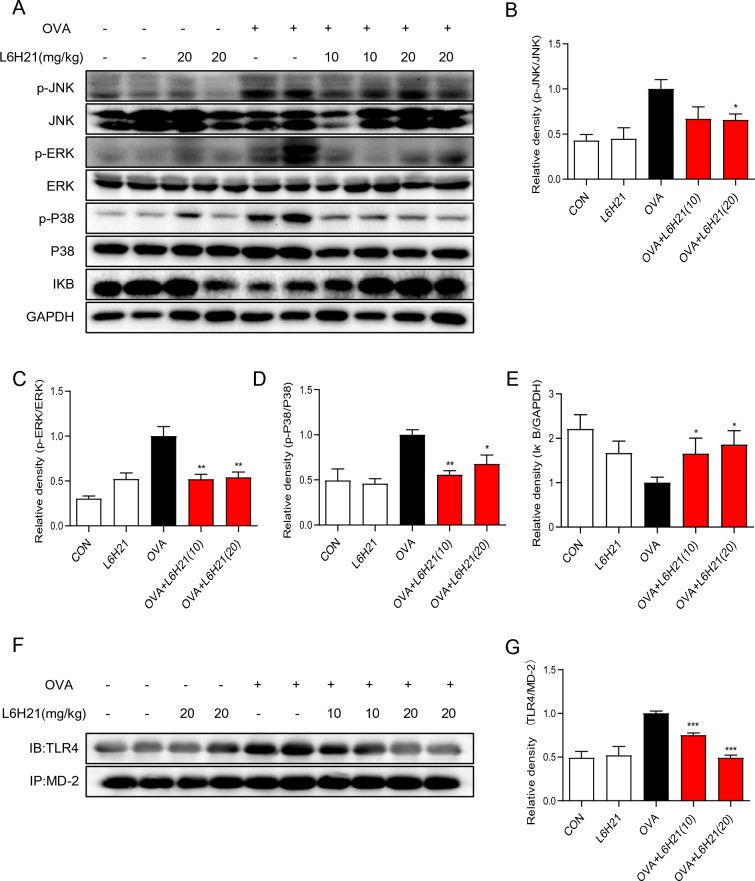
L6H21 attenuated OVA-triggered activation of the MAPK, NF-кB pathways, and combination of TLR4 and MD2 in mouse lung tissue. **A** Part of the lung tissue was collected and homogenated to detect the phosphorylation of P38, ERK, and JNK and the decomposition of IкB by Western blotting assay. **B**–**E** Relative density of P-JNK/JNK (**B**), P-ERK/ERK (**C**), P-P38/P38 (**D**), and IкB/GAPDH (**E**) were obtained. **F** Part of the lung tissue was collected and homogenated to detect the combination of TLR4 and MD2 by co-immunoprecipitation. **G** Relative density of TLR4/MD2 were obtained (results have an expression of the mean ± SEM; *n* = 4 in the respective group; *P < 0.05, **P < 0.01, ***P < 0.001, in comparison with the OVA group)

The downstream signals of TLR4/MD2 primarily consist of the MyD88-dependent MAPKs/NF-κB inflammatory factor transcription pathway and the TRIF-dependent interferon-β (IFN-β) transcription pathway [7]. Several studies have revealed that the transcription and expression of inflammatory cytokines, adhesion molecules and chemokines could be turned on by MAPKs/NF-κB inflammatory factor transcription pathway [9, 52]. Accordingly, the phosphorylation of the MAPK pathway and the NF-кB-signaling pathway activation in mouse lung tissue were examined with the use of Western blotting assay to explore the possible mechanism of L6H21 alleviating asthma. The results unveiled the increased phosphorylation of P38, ERK, and JNK, and the decreased levels of IκB in the OVA group, as well as the decreased phosphorylation of MAPK pathway proteins and the restoration of IκB levels in the L6H21 treatment group (both 10 mg/kg and 20 mg/kg) (Fig. 6A–E).

### L6H21 reduces the OVA-triggered combination of TLR4 and MD2

As reported previously, it is the combination of MD2 and TLR4 that provokes the activation of the downstream MAPK and NF-кB-signaling pathway [53]. Therefore, whether there is increased binding of MD2 and TLR4 in our asthma model was determined through co-immunoprecipitation assay. As illustrated in Fig. 6F, G, the MD2–TLR4 complex formation was increased in the OVA group, while pretreatment with L6H21 could prevent this increase. These results revealed that L6H21 exhibited its protective effects on the asthma model by targeting MD2 and disrupting the TLR4-signaling pathway.

## Discussion

The pathogenesis of asthma exhibits marked heterogeneity with many phenotypes defining visible characteristics and endotypes defining molecular mechanisms. Th1/Th2 imbalances are considered as the underlying mechanisms of classic allergic asthma [29]. An increase in the number of Th2 cells with increased secretion of IL-4, IL-5 and IL-13 may facilitate the production of IgE, proliferation and activation of eosinophils, and secretion of various inflammatory mediators to induce chronic airway inflammation [5]. Due to its central role in the causation of asthma, targeting Th2 induced inflammation seems promising [24]. However, anti-type 2 biological agents are not a panacea for all patients with asthma [16, 37]. Current asthma research is crucially directed towards the development of new and effective drugs, based on anti-inflammatory mechanisms. Scientists have found that genetic variants of MD2 may be associated with an increased risk of asthma [41]. Our own research has demonstrated that the expression level of MD2 is higher in asthma patients than in those without asthma, signifying that treatments focusing on MD2 could be an effective strategy for treating asthma.

Eosinophils are a significant component of the Th2 response in asthma. They may be associated with the causation of classic allergic asthma due to their immunomodulatory and pro-inflammatory functions. Activated eosinophils secrete a variety of cytokines during the development of asthma, including Th2 cytokines (IL-4 and IL-5), acute proinflammatory cytokines (e.g., TNF-α, IL-6, and IL-8) and chemokines [4]. These factors are related to many characteristic features of asthma (e.g., AHR and goblet cell metaplasia) [15]). In this study, the compound L6H21 demonstrated an excellent improvement on OVA-triggered airway inflammation and AHR by ameliorating the infiltration of inflammatory cells (especially eosinophils) and the secretion of Th2 cytokines and acute proinflammatory cytokines.

However, not all patients with typical allergic asthma can obtain complete remission with targeting Th2 cytokine therapy only. Other studies [18, 30, 55] have unveiled that OVA stimulates airway epithelial cells and airway smooth muscle cells to initiate inflammatory signal pathways such as MAPKs and NF-κB and induce various inflammatory factors such as TNF-α and IL-6, leading to a series of destructive pathological changes in airway epithelial cells and airway smooth muscle cells, as well as the exacerbation of asthma. IL-6 is a multi-effect cytokine produced by airway macrophages and bronchial epithelial cells [32, 42]. It can induce antigen-stimulated T lymphocyte proliferation and B lymphocyte maturation, increase IgE secretion, up-regulate local immune responses, and drive the release of systemic acute-phase reactants [31]. Ghaffar reported that airway smooth muscle cells can synthesize and secrete specific eosinophil-activating chemokine (Eotaxin) under TNF-α stimulation, and Eotaxin can provoke an increase in eosinophils, basophils, and helper T cells in the airways of asthmatic patients [10]. Compound L6H21 markedly suppressed the abnormal increase of IL-6 and TNF-α levels in mouse primary macrophages induced by LPS stimulation in our previous in vitro experiments [46]. In our study, TNF-α and IL-6 levels in BALF and lung tissues of the respective group were examined to reveal whether L6H21 reduces IL-6 and TNF-α levels in mouse asthma model, and positive results were identified in the L6H21 treatment group.

TLR4, expressed in myeloid cells and airway epithelial cells [23, 45], may be the main pattern recognition receptor for asthma allergens. TLR4 and its downstream intracellular signaling (mainly involving MAPKs/NF-κB) have been reported to play a certain effect in mediating the occurrence and development of asthma. Interestingly, a clinical study [21] suggested that the single nucleotide polymorphism of MD2, the helper proteins of TLR4, was closely associated with an increased risk of hospital admissions with acute exacerbations. Hosoki discovered that MD2 promoted the development of allergic airway inflammation induced by allergens such as pollen and cat dander [17]. Moreover, L6H21 is not only a compound from natural chalcone with good anti-inflammatory activities but has also been verified as an inhibitor of MD2 in vitro [46]. The TLR4 pathway can be activated and lead to disease exacerbation in the OVA-triggered asthma model [40]. MAPKs/NF-κB inflammatory factor transcription pathway, downstream signals of TLR4/MD2, can turn on the transcription and expression of inflammatory cytokines and chemokines. Hence, it was validated in our study that L6H21 can improve OVA-triggered inflammation through MAPKs/NF-κB pathway (Fig. 6A). Finally, co-immunoprecipitation was adopted to confirm whether L6H21 can affect the complexation of MD2 and TLR4 as an inhibitor of MD2 in vivo. Our data demonstrated that L6H21 dose-dependently affected OVA-triggered MD2 complexing with TLR4. Despite these insights, certain aspects remain unclear, such as the mechanism of the TLR4/MD2 pathway affecting Th2 cytokine secretion, remain to be further explored.

**Fig. 7:**
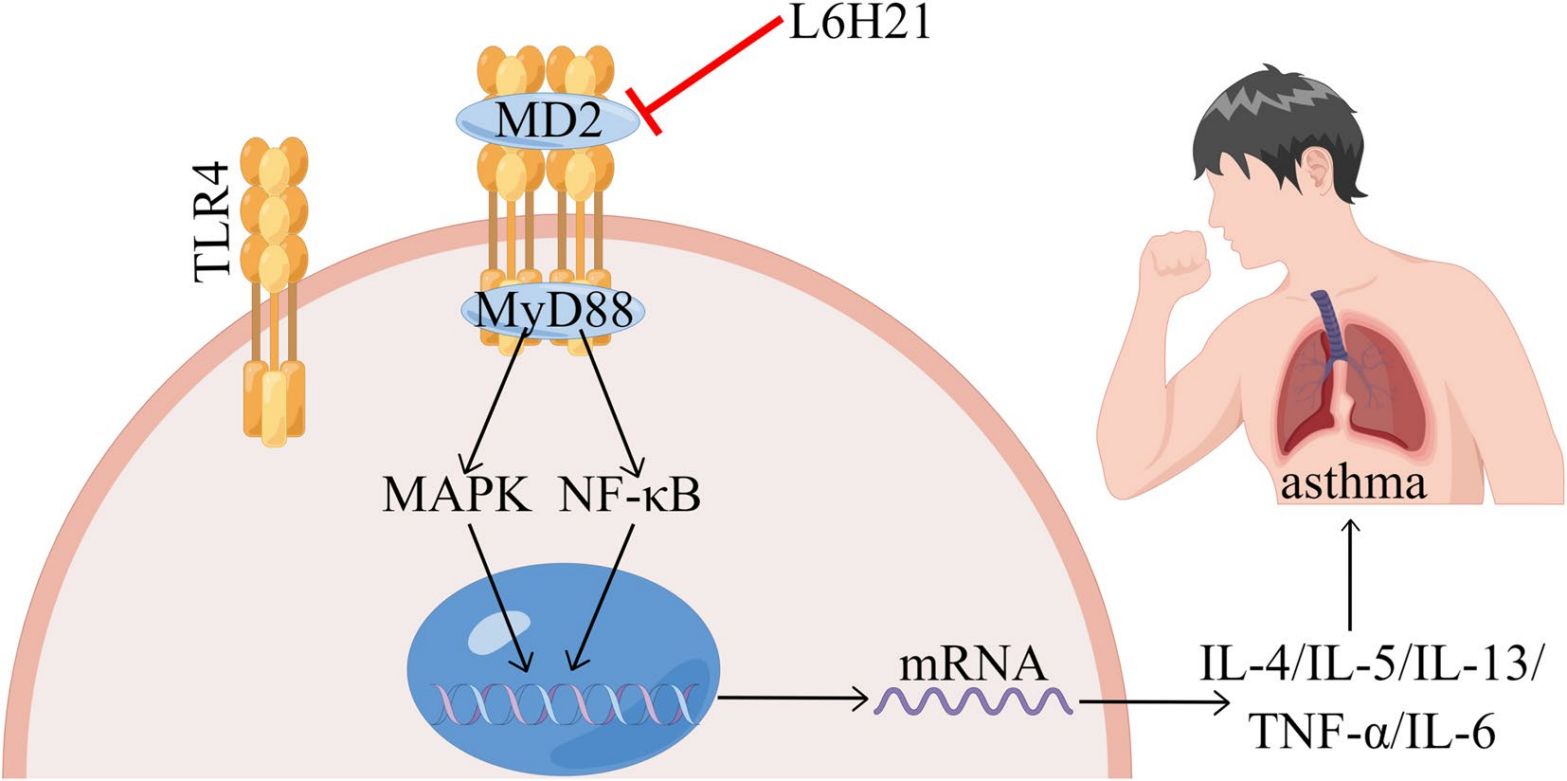
Mechanism diagram of Chalcone derivative L6H21 attenuates the OVA-induced asthma by targeting MD2. [By Figdraw (www.figdraw.com)]

To sum up, L6H21 can dose-dependently reduce the complexation of MD2 and TLR4 induced by OVA, weak MAPKs/NF-κB activation, improve the massive secretion of inflammatory factors and the excessive recruitment of inflammatory cells, and alleviate airway damage (Fig. 7). In other words, MD2 has a great significance in the development of asthma and could serve as a potential target for its treatment with MD2 inhibitors like L6H21, that can be a candidate drug for asthma treatment.

## Data Availability

Not applicable.

## Abbreviations

AHR: Airway hyperresponsiveness
BALF: Bronchoalveolar lavage fluid
DAB: Diaminobenzidine
ECM: Extracellular matrix
H&E: Hematoxylin and eosin
IFN-β: Interferon-beta
IL-6: Interleukin-6
LPS: Lipopolysaccharide
MD2: Myeloid differentiation protein 2
OVA: Ovalbumin
PAS: Periodic Acid Schiff
Rn: Resistance
RT-qPCR: Real-time quantitative polymerase chain reaction
SDS–PAGE: Sodium dodecyl sulfate–polyacrylamide gel
SPF: Specific pathogen free
TLR4: Toll-like receptor 4
UV: Ultraviolet
